# Superior outcomes with Argatroban for heparin-induced thrombocytopenia: a Bayesian network meta-analysis

**DOI:** 10.1007/s11096-021-01260-z

**Published:** 2021-03-28

**Authors:** Giorgia Colarossi, Nicola Maffulli, Andromahi Trivellas, Heike Schnöring, Nima Hatam, Markus Tingart, Filippo Migliorini

**Affiliations:** 1 Department of Cardiac and Thoracic Surgery, University Clinic Aachen, RWTH Aachen University Clinic, Pauwelsstraße 30, Aachen, 52074 Germany; 2 Department of Medicine, Surgery and Dentistry, University of Salerno, Via S. Allende, 84081 Baronissi, SA Italy; 3 School of Pharmacy and Bioengineering, Keele University School of Medicine, Thornburrow Drive, Stoke on Trent, England; 4 Barts and the London School of Medicine and Dentistry, Centre for Sports and Exercise Medicine, Mile End Hospital, Queen Mary University of London, 275 Bancroft Road, London, E1 4DG England; 5 Department of Orthopaedics, David Geffen School of Medicine At UCLA, Los Angeles, CA USA; 6 Department of Orthopaedics and Trauma Surgery, University Clinic Aachen, RWTH Aachen University Clinic, Pauwelsstraße 30, 52074 Aachen, Germany

**Keywords:** Anticoagulants, Bleeding, Heparin-induced thrombocytopenia, Mortality, Thromboembolism

## Abstract

*Background *Argatroban, lepirudin, desirudin, bivalirudin, and danaparoid are commonly used to manage heparin-induced thrombocytopenia related complications. However, the most suitable drug for this condition still remains controversial. *Aim of the review* This Bayesian network meta-analysis study compared the most common anticoagulant drugs used in the management of heparin-induced thrombocytopenia. *Method* All clinical trials comparing two or more anticoagulant therapies for suspected or confirmed heparin-induced thrombocytopenia were considered for inclusion. Studies concerning the use of heparins or oral anticoagulants were not considered. Data concerning hospitalisation length, thromboembolic, major, and minor haemorrhagic events, and mortality rate were collected. The network analyses were made through the STATA routine for Bayesian hierarchical random-effects model analysis with standardised mean difference (SMD) and log odd ratio (LOR) effect measures. *Results* Data from a total of 4338 patients were analysed. The overall mean age was 62.31 ± 6.6 years old. Hospitalization length was considerably shorter in favour of the argatroban group (SMD: − 1.70). Argatroban evidenced the lowest rate of major (LOR: − 1.51) and minor (LOR: − 0.57) haemorrhagic events. Argatroban demonstrated the lowest rate of thromboembolic events (LOR: 0.62), and mortality rate (LOR: − 1.16). *Conclusion* Argatroban performed better overall for selected patients with HIT. Argatroban demonstrated the shortest hospitalization, and lowest rate of haemorrhages, thromboembolisms, and mortality compared to bivalirudin, lepirudin, desirudin, and danaparoid.

## Impacts on practice

- In patients with heparin-induced thrombocytopenia, rapid discontinuation of heparin and transition to a different anticoagulant therapy are essential.
- Several drugs have been proposed for the management of heparin-induced thrombocytopenia; however, the most effective drug remains controversial.
- Anticoagulant therapy in patients with heparin-induced thrombocytopenia reduces thrombotic and hemorrhagic risks.
- While the most effective drug remains controversial, this meta-analysis suggest argatroban may be superior to the other used drugs for the management of heparin induced thrombocytopenia.

## Introduction

Heparin-induced thrombocytopenia (HIT) is an immuno-mediated disorder that occurs in up to 5% of patients receiving therapeutic doses of heparin [1, 2]. In selected patients, heparin exposure induces the formation of IgG-PF4-heparin complex [3] which can promote platelet activation and aggregation [4]. Typically, HIT occurs within ten days after the start of heparin administration [1]. A second exposure to heparin before antibodies have disappeared can result in a rapid onset of HIT [5]. Of note, the incidence of HIT is higher in patients receiving unfractionated heparin than in those exposed to low molecular weight heparin [6]. Low molecular weight heparin induces a smaller complex with PF4 compared to unfractionated heparin [7]. HIT occurs mainly in surgical patients, especially after cardiac or orthopaedic surgery [8]. The diagnosis of HIT is both clinical and serological [9]. ELISA assay and serotonin release assay have high sensitivity but low specificity for HIT [9]. The diagnosis is confirmed by the presence of “HIT antibodies” and any of the following events: (1) unexplained fall in platelet count (< 30% to < 50%); (2) thrombosis; (3) skin lesions at the heparin injection site; (4) acute systemic (anaphylactic) reactions [10]. Up to 55% of patients present deep venous thrombosis, while arterial thromboembolisms are uncommon [11]. Cessation of heparin may not be sufficient to prevent thromboembolic complications; thus, anticoagulant therapy must be promptly initiated [12, 13]. Argatroban and lepirudin are direct thrombin inhibitors (DTI) approved by the Food and Drug Administration (FDA) for the treatment of HIT [14]. Bivalirudin, desirudin, and danaparoid are also commonly used for the management of HIT. Bivalirudin is a synthetic analogue of hirudins indicated for patients with HIT undergoing percutaneous coronary interventions (PCI) [15, 16]. Desirudin is another DTI, belonging to the hirudins family, which is commonly administered in major orthopaedic procedures [17–19]; although danaparoid is a low molecular heparinoid, it also has been commonly administered to prevent complications of HIT [20, 21].

### Aim of the review

Several studies compared the effectiveness of different parenteral anticoagulants for HIT, but the most effective drug for HIT remains controversial [22–40]. The present study compared the most common anticoagulant drugs used for the management of HIT. Therefore, a Bayesian network meta-analysis was conducted. The outcomes of interest of this study were hospitalization length, mortality, haemorrhagic, and thromboembolic rates.

## Method

### Search strategy

This Bayesian network meta-analysis was conducted according to the PRISMA extension statement for reporting of systematic reviews incorporating network meta-analyses of health care interventions [41]. The PICO protocol guided the initial search:

- P (Population): HIT;
- I (Intervention): parenteral anticoagulant therapies;
- C (Comparison): argatroban, bivalirudin, lepirudin, desirudin, danaparoid;
- (Outcomes): hospitalization length, haemorrhages, thromboembolism, mortality.

### Literature search

Two independent reviewers (GC; FM) performed the literature search in February of 2021. The following online databases were accessed: Pubmed, EMBASE, Google Scholar, Scopus. The following keywords were used in combination: *HIT, heparin, induced, thrombocytopenia, thromboembolism, deep vein thrombosis, bleeding, haemorrhagic, anticoagulants, argatroban, bivalirudin, lepirudin, desirudin, danaparoid, hospitalization length, platelet, haemoglobin, haematocrit, blood, thromboembolism, death, survivorship, therapy, treatment, thrombin, thrombin inhibitor, thrombosis prophylaxis, PF4, PF4 antibodies*. The resulting articles were screened for inclusion. If the title and abstract matched the topic, the full text article was accessed. The bibliographies of the included studies were also screened for inclusion.

### Eligibility criteria

All clinical trials investigating parenteral anticoagulant therapies for suspected or proven HIT were considered for inclusion. Studies in English, Italian, German, French, and Spanish, according to the authors language capabilities, were included. Studies of level of evidence I to III, according to the Oxford Centre of Evidenced-Based Medicine [42], were considered. Reviews, comments, editorials, opinions, reports, and data from registries were not considered. Animal and cadaveric studies were not included. Studies using other drugs rather than parenteral anticoagulants were not included. Trials comparing anticoagulants with heparin were not included, nor were studies that evaluated the effectiveness of these compounds in other thrombocytopenia conditions (e.g. neoplastic, idiopathic, haemolytic). Articles performing studies on oral anticoagulants (e.g. vitamin K antagonists, direct oral anticoagulants) were not included. Only clinical trials reporting quantitative data under the outcomes of interest were considered for inclusion. Disagreements between the reviewers were debated and solved by a third author (SH).

### Outcomes of interest

Data extraction was performed by two independent reviewers (GC, FM). Generalities of the studies were collected (author and year, journal, study design, eligibility criteria). Patient baseline was extracted, along with the name, dose, therapeutic and maintenance doses for each drug. The length of hospitalization was retrieved. Data on the rate of major and minor haemorrhagic events, thromboembolic complications, and mortality were collected.

### Methodological quality assessment

The methodological quality assessment was performed by one reviewer (GC). For this purpose, the bias summary tool of the Review Manager Software (The Nordic Cochrane Collaboration, Copenhagen) was used. The following biases were analysed: selection, detection, attrition, reporting, and other sources of bias.

### Statistical analysis

The statistical analyses were performed by the senior author (FM). For baseline comparability, the IBM SPSS software was used. Comparability was assessed through the Analysis of Variance (ANOVA), with *P* > 0.1 considered satisfactory. The network analyses were made through the STATA/MP software (Stata Corporation, College Station, Texas, USA). The analyses were performed through the Stata routine for Bayesian hierarchical random-effects model analysis. Continuous variables were analysed through the inverse variance method, with the standardized mean difference (SMD) effect measure. Binary data was analysed through the Mantel–Haenszel method, with the Log Odd Ratio (LOR) effect measure. Edge, interval, and funnel plots were performed and analysed. The overall transitivity, consistency, and heterogeneity, as well as the size of the treatment effect of interest within-study variance, were evaluated. The overall inconsistency was evaluated through the equation for global linearity via the Wald test. If P_Wald_ values > 0.05, the null hypothesis could not be rejected, and the consistency assumption could be accepted at the overall level of each treatment. Confidence and percentile intervals (CI, PrI, respectively) were each set at 95%.

## Results

### Search result

**Fig. 1:**
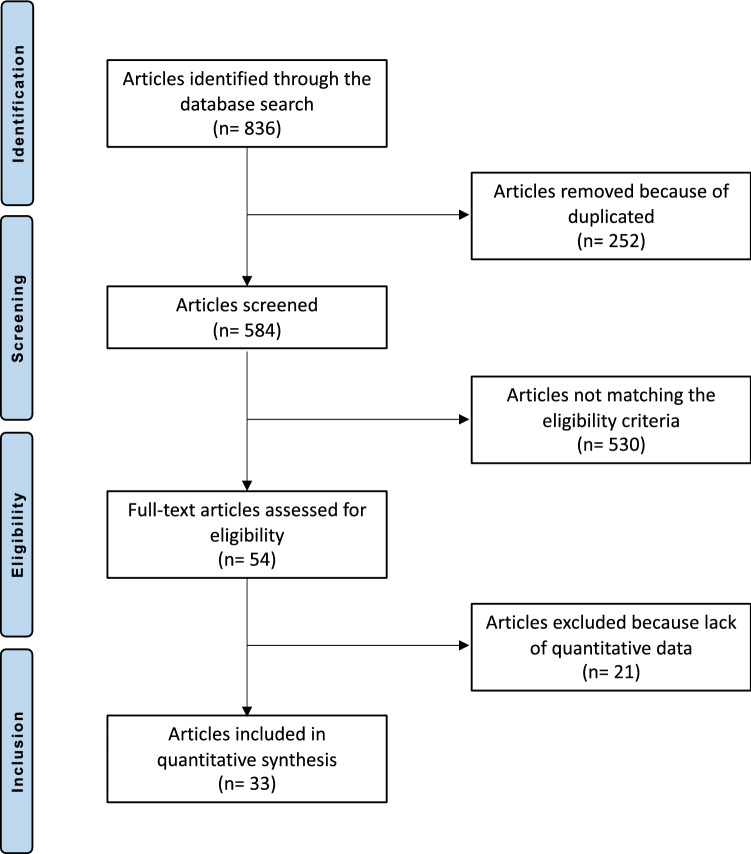
Flow-chart of the literature search

The literature search resulted in 836 articles. Of them, 252 were duplicates. An additional 530 articles were excluded: nature of the study (N = 207), non-clinical studies (N = 153), use of other anticoagulants (N = 84), use of adjuvant(s) (N = 72), language limitations (N = 12), uncertain results (N = 2). Another 21 articles were rejected as quantitative data under the outcomes of interests were missing. Ultimately, 33 articles were included: 4 randomized clinical trials, 16 prospective, and 13 retrospective clinical studies (Fig. 1).

### Methodological quality assessment

**Fig. 2:**
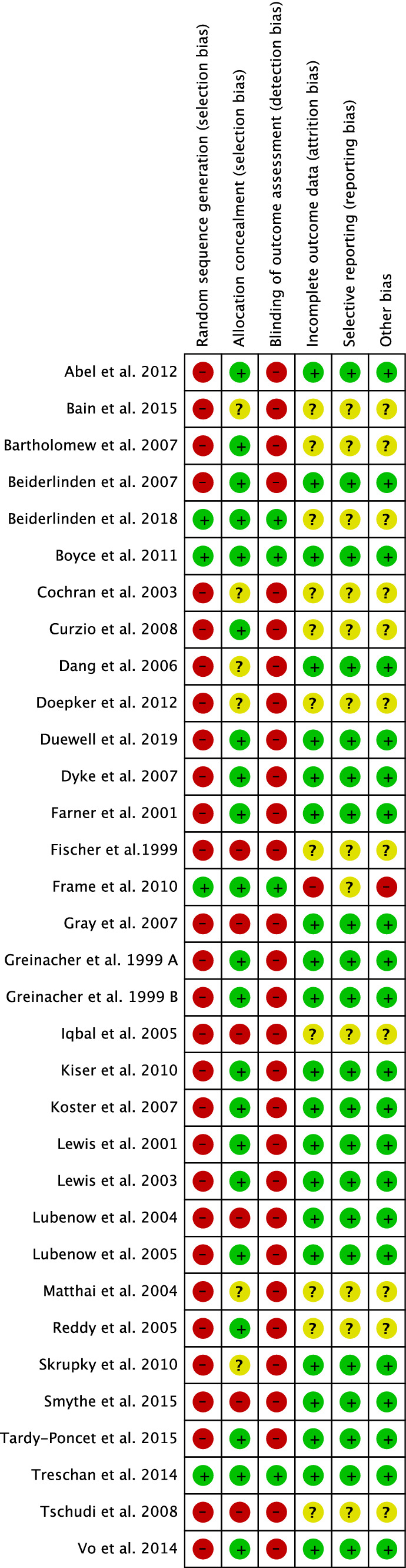
Methodological quality assessment

The Cochrane bias of summary tool evidenced some limitations of the present study. First, the overall retrospective nature of the included studies, along with the overall lacking of blinding methods led to increased selection and detection biases. Indeed, only 12% (4 or 33 studies) were RCTs, and only two of these were blinded. The overall risks of attrition, reporting, and unknown sources of bias were acceptable. In conclusion, the risk of bias of the present work was fair-moderate. The Cochrane bias of summary tool is shown in Fig. 2.

### Patient demographics

Data from 4338 patients were retrieved. A total of 1846 patients were analysed in the argatroban group, 318 in the bilivarudin group, 973 in the lepidurin group, 68 in the desirudin group, and 126 in the danaparoid group. 53.92% (2339 of 4338 patients) were men. The overall mean age was 62.31 ± 6.6 years old. The ANOVA test found good comparability concerning age (P = 0.07). The generalities and demographics of the included studies are shown in Table 1.

**Table 1:** Generalities and baseline characteristic of the included studies

Author, year	Journal	Type of study	Inclusion criteria	Exclusion criteria	Samples	Mean age	Drug
Abel et al. 2012 [59]		RCS	(1) continuous or intermittent RRT and Argatroban, Bivalirudin, or Lepirudin; (2) exposition to heparin within 100 days; (3) documented heparin allergy; (4) absolute platelet count < 150,000 cells/µL and /or platelets decline > 50%	(1) age < 18; (2) receiving bivalirudin for PCI	102	62	Argatroban
	*Am J Health-Syst Pharm*				18	54	Bivalirudin
					41	61	Lepirudin
Bain et al. 2015 [28]	*Am J Health-Syst Pharm*	RCS	(1) age > 18; (2) DTI treatment for suspected HIT	(1) receiving bivalirudin for acute coronary syndrome; (2) treatment duration < 12 h	9	68	Argatroban
					14	61	Bivalirudin
					25	66	Lepirudin
Bartholomew et al. 2007 [60]	*Drugs Aging*	RCS	(1) age ≥ 65; (2) platelet count < 50% during heparin treatment (3) heparin-PF4 antibodies positive (ELISA); (4) previous HIT after previous heparin exposure		62		Argatroban
Beiderlinden et al. 2007 [29]	*Ann Pharmacother*	PCS	(1) platelet count < 50%; (2) minimum 2 organ failures according to SOFA score	(1) intracranial surgery; (2) transient thrombocytopenia due to intraoperative and/ or active bleeding; (3) spontaneous aPTT > 70 s; (3) age < 19; (4) pregnancy; (5) chronic hepatopathy	5	49	Argatroban
					19	51	Argatroban
Beiderlinden et al. 2018 [61]	*BMC Anesthesiol*	RCT	(1) ICU > 24 h; (2) age > 18; (3) platelet < 50%; (4) persisting > 24 h; (5) 4 T-Score > 3 (6) heparin-PF4 antibodies positive (ELISA)	(1) active bleeding; (2) intracranial surgery; (3) aPTT > 60 s; (4) pregnancy	17	72	Argatroban
					18	59	Lepirudin
Boyce et al. 2011 [35]	*Am J Ther*	RCT	(1) age > 18; (2) HIT with or without thrombosis; (3) exposition within the preceding 100d to heparin; (4) rapid platelet count of < 30%; (5) development of skin lesions or an acute systemic reactionary to heparin; (6) estimated survival > 30d	(1) pregnancy; (2) suspected or confirmed pulmonary embolism or acute ischemic stroke; (3) cerebrovascular accident within 6 months Intracranial neoplasm, arteriovenous malformation, or aneurysm; (4) severe renal insufficiency; (5) hirudin assumption within 6 m before enrollment; (6) > 2 doses of fondaparinux for treatment of suspected HIT; (7) estimated survival < 30d; (8) uncontrolled hypertension; (9) requirement for indwelling mechanical intervention	8	62	Argatroban
					8	69	Desirudin
Cochran et al. 2003	*J Invasive Cardiol*	PCT			25		Lepirudin
Curzio et al. 2008 [62]		RCS	(1) heparin-PF4 antibodies positive (ELISA); (2) age > 18; (3) platelet count < 50% during the first 30d	(1) subjects that concurrently received abciximab during hospitalization	17	68	Argatroban
	*J Thromb Thrombolysis*				24	63	Lepirudin
					41	68	none
Dang et al. 2006 [38]		RCS	(1) age > 18; (2) direct thrombin inhibitor for treatment of anticoagulation for > 24hs during their hospital stay	(1) treatment solely for percutaneous coronary intervention	13	52	Argatroban
	*Pharmacotherapy*				24	52	Bivalirudin
					5	67	Lepirudin
Doepker et al. 2012 [33]	*J Thromb Thrombolysis*	RSC	(1) age 18 to 89	(1) pregnancy	73	59	Argatroban
Duewell et al. 2019 [26]	*J Pharm Pract*	RCS	(1) age > 18; (2) heparin-PF4 antibodies positive (ELISA) (3) Argatroban or Bivalirudin for > 6 h	(1) DTI therapy for an indication other than suspected or confirmed HIT	45	61	Argatroban
					46	60	Bivalirudin
Dyke et al. 2007 [63]	*Ann Thorac Surg*	PCS	(1) age > 18; (2) off-pump coronary artery bypass	(1) severe renal dysfunction; (2) severe left ventricular dysfunction; (3) recent stroke	51	64	Bivalirudin
Farner et al. 2001 [53]		PCS	(1) platelet count < 50% (2) platelet values < 100 × 10^6^ mL (3) thromboembolic complications (4) heparin-PF4 antibodies positive (ELISA)	(1) no definite need for parenteral anticoagulation (other than HIT) according to the judgment of the treating physician; (2) abuse of alcohol/ drugs; (3) pregnancy; (4) renal impairment; (5) age under 18; (6) overt bleeding or enhanced bleeding risk at diagnosis of HIT; (7) cardiopulmonary-bypass surgery during the respective hospital period	126	65	Danaparoid
	*Thromb Haemost*				115	58	Lepirudin
					60	58	Lepirudin
Frame et al. 2010 [34]	*Clin Ther*	RCT	(1) age > 18; (2) with or without thrombosis; (3) received heparin within the previous 100d (4) platelet count < 30% within 24 h	(1) pregnancy; (2) pulmonary embolism requiring continued anticoagulation or acute ischemic stroke; (3) cerebrovascular accident within 6 m (4) Intracranial neoplasm, arteriovenous malformation, or aneurysm; (5) severe renal insufficiency; (6) administration of hirudin within 6 m before enrollment; (7) ≥ 2 doses of fondaparinux for treatment of suspected HIT; (8) estimated survival < 30d; (9) active bleeding or irreversible coagulation abnormality; (10) uncontrolled hypertension; (11) requirement for indwelling mechanical intervention; (12) uncontrolled severe disease	120		Argatroban
							Desirudin
Fischer et al. 1999 [40]	*Kidney Int Suppl*	PCS	(1) continuous renal replacement therapy		7	57	Lepirudin
Gray et al. 2007 [32]	*Clin Appl Thromb Hemost*	RCS	(1) platelet decrease > 50%; (2) platelet count < 100 × 10^6^ mL		390	64	Argatroban
					98	66	HCT
Greinacher et al.1999 [30]	*Circulation*	PCS	(1) age > 18; (2) platelet count < 100 × 106/mL; (3) platelet count < 50%; (4) TECs during heparin therapy; (5) history of HIT	(1) missing date of laboratory confirmation of HIT; (2) time between clinical symptoms and laboratory confirmation > 21 days; (3) time between laboratory confirmation and initiation of therapy > 60 days; (4) cardiopulmonary bypass; (5) alcohol or drug abuse; (6) bleeding; (7) pregnancy; (8) poor compliance	65	56	Lepirudin
					4	44	Lepirudin
					43	62	Lepirudin
					120	67	HCT
Greinacher et al.1999 [64]		PCS	(1) age > 18; (2) platelet count < 100 × 10^6^ mL; (3) platelet count decrease > 30%; (4) TECs during heparin therapy; (5) history of HIT	(1) hemodialysis or hemofiltration; (2) hypersensitivity to hirudin; (3) pregnancy; (4) poor compliance	51	60	Lepirudin
					5	41	Lepirudin
	*Circulation*				18	61	Lepirudin
					8	72	Lepirudin
					120	65	HCT
Iqbal et al. 2005 [36]		PCS	(1) off-pump coronary artery revascularization		1	57	Lepirudin
	*J Card Surg*				1	49	Lepirudin
					1	70	Lepirudin
Kiser et al. 2010	*Am J Hematol*	PCS	(1) age > 18; (2) heparin-PF4 antibodies problems (ELISA)	(1) DTI for percutaneous coronary intervention or cardiopulmonary bypass; (2) goal aPTT range not 1.5–2.5 times baseline aPTT or the upper limit of the normal aPTT laboratory range	47	56	Argatroban
							Bivalirudin
					83	52	HCT
							HCT
Koster et al. 2007 [65]	*Ann Thorac Surg*	PCS	(1) age > 18; (2) CABG single valve surgery; (3) CABG plus single-valve surgery	(1) severe renal dysfunction; (2) ventricular ejection fraction < 0.30; (3) required surgery on more than one heart valve; (4) recent stroke, or with a residual neurologic deficit	50		Bivalirudin
Lewis et al. 2003 [66]	*Arch Intern Med*	PCS	(1) age > 18; (2) platelet count < 100 × 106/mL; (3) platelet count < 50%	(1) unexplained aPTT > 2 × the baseline value; (2) documented coagulation disorder or bleeding diathesis unrelated to HIT; (3) lumbar puncture in the past 7d; (4) history of previous aneurysm, hemorrhagic stroke, or recent thrombotic stroke; (5) known bleeding site; (6) current pregnancy or breastfeeding; (7) life expectancy of < 2w	189	64	Argatroban
					229	64	Argatroban
					139	66	HCT
					46	66	HCT
Lewis et al. 2001 [39]	*Circulation*	PCS	(1) age 18 to 80; (2) platelet count < 100 × 10^6^ mL (3) platelet count < 50%; (4) documented history of HIT-Ab + who required anticoagulation, in the absence of thrombocytopenia or heparin challenge	(1) unexplained aPTT > 2 × baseline; (2) documented coagulation disorder or bleeding diathesis unrelated to HIT; (3) lumbar puncture within the past 7d; (4) history of previous aneurysm, hemorrhagic stroke, or recent (within 6 months) thrombotic stroke unrelated to HIT	160	61	Argatroban
					144	62	Argatroban
					147	66	HCT
					46	66	HCT
Lubenow et al. 2005 [67]	*J Thromb Heamost*	PCS	(1) age > 18; (2) platelet count < 100 × 106/mL; (3) platelet count < 50%; (4) TECs during heparin therapy; (5) history of HIT	(1) treatment with any other investigational drug within 7 days before study entry; (2) alcohol or drug abuse; (3) bleeding; (4) hypersensitivity to hirudin; (5) pregnancy; (6) poor compliance	98	61	Lepirudin
					12	57	Lepirudin
					84	65	Lepirudin
					10	69	Lepirudin
					120	67	HCT
Lubenow et al. 2004 [27]	*Blood*	PCS	(1) platelet count < 100 × 106/mL; (2) platelet count decrease > 30%; (3) positive heparin-induced platelet activation (HIPA) test; (4) no clinically evident thrombosis		91	63	Lepirudin
					47	66	HCT
Matthai et al. 2004 [31]	*Thromb Res*	PCS	(1) history of HIT, who required anticoagulation; (2) prior episode of HIT serologically confirmed; (3) platelet count > 150 × 106/mL	(1) unexplained coagulopathy or documented coagulation disorder; (2) increased bleeding risk	36	67	Argatroban
Reddy et al. 2005 [24]	*Ann Pharmacother*	RSC	(1) platelet count < 100 × 106/mL; (2) platelet count < 50%; (3) TECs during heparin therapy; (4) history of HIT	(1) unexplained coagulopathy; (2) documented coagulation disorder; (3) increased bleeding risk	47	69	Argatroban
Skrupky et al. 2010 [37]	*Pharmacotherapy*	RSC	(1) age > 18; (2) receiving either Argatroban or Bivalirudin between January 2007 and July 2008 for > 24hs; (3) known or suspected HIT		46	62	Argatroban
					92	57	Bivalirudin
Smythe et al. 2015 [68]	*Clin Appl Thromb Hemost*	RSC		(1) PCI	29	69	Argatroban
					61	68	Lepirudin
Tardy-Poncet et al. 2015 [69]	*Crit Care*	PCS	(1) age > 18; (2) heparin-PF4 antibodies positive (ELISA, PAT or SRA); (3) parenteral anticoagulation		20	72	Argatroban
Treschan et al. 2014 [52]	*Crit Care*	RCT	(1) ICU > 24 h; (2) age > 18; (3) platelet count < 50% (4) symptoms > 24 h; (5) 4 T-Score > 3 (6) heparin-PF4 antibodies positive (ELISA)	(1) active bleeding; (2) intracranial surgery; (3) aPTT > 60 s; (4) pregnancy	34	68	Argatroban
					32	64	Lepirudin
Tschudi et al. 2009 [51]	*Blood*	RSC			68	69	Lepirudin
Vo et al. 2014 [23]	*Ann Pharmacother*	RSC	(1) age > 18; (2) > 24 h of Argatroban or bivalirudin for suspected HIT	(1) treatment initiated outside of the institution (2) Argatroban or bivalirudin received within 30d prior to DTI initiation; (3) no aPTT goal documented, (4) DTI treatment prescribed for indications other than HIT	48	70	Argatroban
					20	64	Bivalirudin

RCS: retrospective cohort study; RCT: randomised controlled trial; PCS: prospective cohort study; HCT: historical control therapy; RRT: renal replacement therapy; PCI: percutaneous coronary intervention; SOFA: sequential organ failure assessment; ELISA: enzyme-linked immunosorbent assay; aPTT: activated partial thromboplastin time; TECs: thromboembolic complications; CABG: coronary artery bypass graft; HIPA: heparin-induced platelet-activation test; PAT: platelet aggregation test; SRA: serotonin release assay; DTI: direct thrombin inhibitor

### Outcomes of interest

**Fig. 3:**
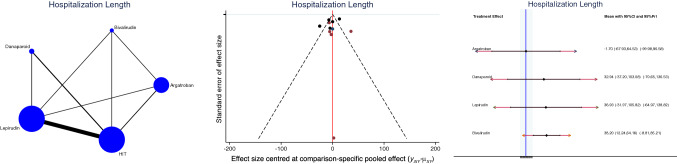
Edge, funnel, and interval plots of the network comparison: hospitalization length

Hospitalization length was shorter in the argatroban group (SMD: − 1.70; 95% CI: − 67.93 to 64.53; Fig. 3).

**Fig. 4:**
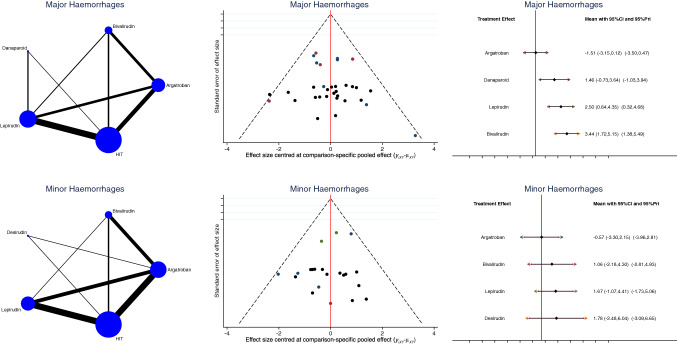
Edge, funnel, and interval plots of the network comparisons: major and minor haemorrhagic events

Argatroban demonstrated the lowest rate of major (LOR: − 1.51; 95% CI: − 3.15 to 0.12; Fig. 4) and minor (LOR: − 0.57; 95% CI: − 3.30 to 2.15; Fig. 4) haemorrhagic events.

**Fig. 5:**
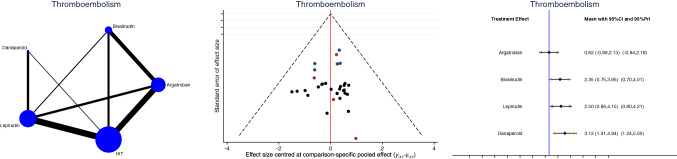
Edge, funnel, and interval plots of the network comparison: thromboembolic events

**Fig. 6:**
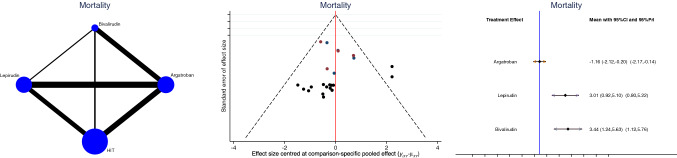
Edge, funnel, and interval plots of the network comparison: mortality rate

Argatroban demonstrated the lowest rate of thromboembolic events (LOR: 0.62; 95% CI: − 0.89 to 2.13; Fig. 5) and mortality rate (LOR: − 1.16; 95% CI: − 2.12 to − 0.20; Fig. 6).

The equation for global linearity found no evidence of inconsistency among the comparisons: hospitalization (*P* = 0.3), major (*P* = 0.2) and minor (*P* = 0.4) haemorrhagic events, thromboembolism (*P* = 0.3), mortality rate (*P* = 0.2).

## Discussion

According to the main findings of this Bayesian network meta-analysis, argatroban performed better than bivalirudin, lepirudin, desirudin, danaparoid for selected patients with HIT. Argatroban demonstrated the lowest rate of haemorrhages, thromboembolisms, and mortality, along with the shortest length of the hospitalization, compared to the other drugs of interest.

HIT is an immune mediated reaction triggered by the exposure to unfractionated heparin or low molecular weight heparin. Clinical manifestation is typically seen in 5 to 10 days after the start of heparin therapy [5, 43]. The most severe complications of HIT are thromboembolic events, and several studies have reported that heparin cessation may not be enough to prevent thrombosis: another anticoagulation method is recommended [44, 45]. Hirsh et al. [4] investigated the treatment of HIT with argatroban, lepirudin, and danaparoid. Despite the use of another anticoagulation method, the mortality in HIT is up to 22%, while the risk of a new thromboembolic event is up to 18%. In the present study, the efficacy and safety of five different anticoagulants were analysed: four belonging to the DTI family (argatroban, lepirudin, desirudin, bivalirudin) and one low molecular weight heparinoid (danaparoid). Argatroban is a univalent DTI that binds thrombin, inhibiting its action. Its good tolerability and short half-life make argatroban an attractive option for the management of HIT complications [46]. However, given its hepatic metabolism, argatroban is not recommended for patients with hepatic dysfunction [47], unless meticulous dose adjustments and aPTT monitoring are undertaken [44]. Conversely, danaparoid has longer half-life than argatroban, and good efficacy in maintaining stable anticoagulation [48]. Moreover, despite danaparoid being a low-molecular-weight heparinoid, its cross-reactivity with HIT antibodies is rare [48]. Hirudins (lepirudin, desirudin) are bivalent DTI, that bind with a high affinity and specificity to two distinct sites of thrombin [49]. However, give its renal metabolism, lepirudin must be administered with caution in nephropathic patients. Furthermore, immunogenicity has been associated with lepirudin consumption [50, 51]. In a meta-analysis by Greinacher et al. [43], lepirudin demonstrated lower mortality rate and thromboembolic events compared to the control group. Treschan et al. [52] compared argatroban versus lepirudin on 28 patients requiring renal replacement therapy. Patients randomized to lepirudin experienced a greater incidence of haemorrhagic complications [52]. These results were confirmed by the present study, which found a greater rate of haemorrhagic events following administration of lepirudin compared to argatroban and danaparoid. In a retrospective analysis comparing danaparoid (126 patients) versus lepirudin (175 patients) [53], interestingly, danaparoid was associated with a greater risk of new thromboembolic events in patients without thrombosis on admission. Although bivalirudin is mainly metabolized in the liver, nephropathic patients require dose adjustment [38, 54, 55]. Bivalirudin and argatroban were compared in a retrospective study which found that both anticoagulants reached the aPTT goal within six hours, and evidenced similarity between the two compounds [37]. Lastly, a meta-analysis, demonstrated similar efficacy and safety between argatroban, lepirudin, and bivalirudin [56].

This network meta-analysis has certain limitations. The retrospective nature of most of the included studies increased the risk of selection bias, reducing the reliability *of the conclusions of the present study.* The current literature lacks high-quality studies; therefore, additional studies should tackle this limitation. Furthermore, the low number of included studies and related patients represents another limitation. The presence of HIT-related complications on admission were not analysed in the present study, along with patient comorbidities (e.g. patients with liver and/or renal dysfunctions). Given the lack of quantitative data, a subgroup analysis according to the initial and maintenance doses could not be performed. Parenteral anticoagulants are frequently chosen for the management of HIT [22, 57]. While many options for anticoagulant therapy are available, the present study focused on the effects of parenteral anticoagulation. Further analyses concerning the effects of direct oral anticoagulants and vitamin K antagonists for HIT are required. Lastly, fondaparinux is a synthetic heparin polysaccharide, [58] which has not been considered in this study. Given the lack of quantitative data, fondaparinux was not included in the network comparisons. Considering these limitations, data from the present Bayesian network meta-analysis must be interpreted with caution.

## Conclusion

Argatroban performed better overall for selected patients with HIT. Argatroban demonstrated the shortest hospitalization, and lowest rate of haemorrhages, thromboembolisms, and mortality compared to bivalirudin, lepirudin, desirudin, and danaparoid.

## Declarations

Filippo Migliorini and Giorgia Colarossi performed the literature search. Nicola Maffulli and Andromahi Trivellas performed revision and approved the final version. Heike Schnöring, Nima Hatam, and Markus Tingart supervised the research and approved the final version. Filippo Migliorini performed the statistical analyses.
